# Detectable ctDNA at the time of treatment cessation of ipilimumab and nivolumab for toxicity predicts disease progression in advanced melanoma patients

**DOI:** 10.3389/fonc.2023.1280730

**Published:** 2023-12-19

**Authors:** Lydia Warburton, Anna Reid, Benhur Amanuel, Leslie Calapre, Michael Millward, Elin Gray

**Affiliations:** ^1^ Centre for Precision Health, Edith Cowan University, Joondalup, WA, Australia; ^2^ Department of Medical Oncology, Fiona Stanley Hospital, Murdoch, WA, Australia; ^3^ School of Medical and Health Sciences, Edith Cowan University, Joondalup, WA, Australia; ^4^ Anatomical Pathology, PathWest, Queen Elizabeth II (QEII) Medical Centre, Nedlands, WA, Australia; ^5^ School of Biomedical Science, University of Western Australia, Crawley, WA, Australia; ^6^ School of Medicine, University of Western Australia, Crawley, WA, Australia

**Keywords:** immunotherapy, toxicity, biomarkers, CtDNA, melanoma, therapy cessation

## Abstract

**Background:**

Immune checkpoint inhibition (ICI) has led to unprecedented outcomes for melanoma patients but is associated with toxicity. ICI resumption after high grade irAEs poses a significant challenge in the clinical management of melanoma patients and there are no biomarkers that can help identify patients that might benefit from resuming treatment. This study aims to determine if circulating tumor DNA (ctDNA) levels at the time of treatment-limiting irAE could guide treatment decisions in this clinical context.

**Methods:**

This is a retrospective exploratory biomarker study from 34 patients treated with combination ICI for stage IV melanoma. Patients had a treatment-limiting toxicity and a baseline plasma collection prior to commencing ICI and within 6 weeks of stopping therapy. Blood samples were tested for ctDNA at baseline and cessation therapy.

**Results:**

Median progression free survival (PFS) and overall survival (OS) have not been reached (24-month PFS rate 54% and OS rate 72.3%). PD occurred in 47% (16/34) of patients. Median PFS with detectable ctDNA from plasma collected at the time of toxicity was 6.5 months while not reached (NR) with undetectable levels (HR: 4.0, 95% CI 0.95-17.5, p=0.0023). Median OS with detectable ctDNA at cessation for toxicity was 19.4 months and NR for undetectable ctDNA (HR: 3.9, 95%CI 20.8-18.6, p=0.024). Positive ctDNA at the time of cessation was highly specific (specificity 0.94, 95% CI 0.74-0.99, PPV 0.88, 95% CI 0.53-0.99). However, ctDNA negativity has low sensitivity as a predictor of ongoing disease control (sensitivity 0.437, 95% CI 0.23-0.67). Notably, 4/9 (44%) ctDNA negative patients who had disease progression had brain only disease progression.

**Conclusions:**

Undetectable ctDNA and CR on imaging after stopping immunotherapy for toxicity results in high rates of long-term durable control. For patients with immunotherapy related toxicity, who have persistent ctDNA at 8 – 12 weeks, the risk of disease progression is significant.

## Introduction

Combination immunotherapy with anti-CTLA4 and anti-PD1 blockade has led to unprecedented outcomes for advanced melanoma patients. The CheckMate 067 trial was practice-changing and ultimately led to improvements in survival for advanced melanoma (1). Recent data from CheckMate 067 reported 49% of patients randomized to the combination arm were alive at the 6.5 year landmark, with over a third of patients alive and without disease progression at this data cut-off (2). Median OS for this combination has now been reported as 72.1 months. Thus, this combined immunotherapy regimen appears to be highly effective in providing long-term disease control for approximately 50% of patients with advanced melanoma.

This revolutionary achievement does come with the cost of additional toxicity. Immune-related adverse events (irAE) were reported in 96% of patients who received the combination arm in CheckMate 067 (1). The most frequent high grade immune related adverse events were diarrhea, colitis and hepatitis. Treatment related adverse events that led to discontinuation of treatment were reported in over a third of patients. Interestingly, irAE in the treatment of melanoma and lung cancer have been reported to be associated with improved survival (3–6). Currently, clinical decisions about re-challenge with immune checkpoint inhibitors (ICIs) after high grade toxicity are made on the basis of several factors including the type of irAE, the severity of irAE, the tumor response to initial induction treatment, the availability of subsequent lines of treatment and the patient’s ability to tolerate a taper of immunosuppression. The benefit of resumption of ICI after initial toxicity can be significant in select patients (7). ICI resumption after high grade irAEs poses a significant challenge in the clinical management of melanoma patients and currently there are no biomarkers that can help identify patients that might benefit from resuming treatment and those that will have flare of toxicity without any clinical benefit.

Circulating tumor DNA (ctDNA) are fragmented DNA released into plasma from apoptosing or necrotic tumor cells that have emerged as a biomarker for assessment of response to therapy and detection of minimal residual disease in melanoma (8–12). Baseline ctDNA levels have been shown to be directly associated with radiological tumor burden and inversely associated with response and PFS (11). Undetectable ctDNA at baseline or within eight weeks of commencing immunotherapy is an independent predictor of response, PFS and OS (13, 14). This correlation between ctDNA and treatment response could be monopolized to determine the extent of clinical benefit from ICIs in patients that developed irAEs and guide treatment resumption decisions.

This study aims to determine if ctDNA levels at the time of treatment limiting irAE could inform outcomes and guide treatment decisions in this population. This real-world retrospective cohort examines plasma ctDNA from 34 melanoma patients treated with ipilimumab and nivolumab that developed high grade toxicity resulting in discontinuation of treatment. We hypothesized that ctDNA positivity at the time of toxicity might be predictive of disease progression and could eventually be used as a surrogate to determine whether a switch in therapy for non-response based ctDNA dynamics or rechallenge with ICI monotherapy (associated with the potential risk of flare of irAEs) would benefit the patient.

## Materials and methods

### Patients

This retrospective exploratory biomarker study included 68 plasma samples from 34 patients treated with combination immunotherapy for stage IV melanoma (**Supplementary Figure 1**). Initially a total of 135 patients treated with ipilimumab and nivolumab for stage IV melanoma were enrolled in the study between 2013 and 2021 at Sir Charles Gairdner Hospital and Fiona Stanley Hospital in Perth, Western Australia. Patients from this initial cohort were included based on the following criteria: i) had a treatment limiting toxicity defined as a grade III/IV immune related adverse event as per Common Terminology Criteria for Adverse Events (CTCAE v5) documented in the medical record; ii) had a baseline plasma collection prior to commencing doublet immunotherapy and a blood sample collected within 6 weeks of stopping therapy due to toxicity. This study received approval from the Human Research Ethics Committee of Edith Cowan University (Nos. 11543 and 18957) and Sir Charles Gairdner Hospital (No. 2013–246 and RGS0000003289). Written consent was obtained from all patients under approved human research ethics committee protocols that complied with the Declaration of Helsinki.

### Treatment response and disease progression assessment

Radiologic assessment of treatment response and disease progression was performed at the cessation of the combination of ipilimumab and nivolumab following toxicity. This was predominantly done by 18F-labeled fluorodeoxyglucose positron emission tomography (FDG-PET) scans. MRI of the brain was also used where indicated. Patients were considered to have progressive disease (PD) if they developed new lesions, had a significant increase in tumor size as per iRECIST on CT, or presented a new or enlarging clinical lesion, as per the medical imaging reported findings, confirmed by the treating clinician and confirmed on repeat imaging after 4-12 weeks as per iRECIST. Confirmation of disease progression on repeat imaging occurred if.

1. a further increase of the lesions (≥ 5mm increase),
2. a significant increase of a non-target lesion previously classified as iuPD,
3. an increase in the size (≥ 5mm) of a previously new lesion, or
4. the appearance of new lesions.

When PD was confirmed on repeat scans post initial disease progression, the first date of PD was used as the event date for the progression-free survival (PFS) assessment.

Clinicians were blinded to the ctDNA result at the time of the scan. PFS was defined as the time interval between the start of combination immunotherapy and the date of first clinical or radiologic progression or death. OS was defined by the time interval between the start of therapy and death from any cause. Patients alive at the last follow up were censored. Response rates were analyzed at the time of treatment cessation due to toxicity and do not uniformly represent best response. Objective response rates were defined as the percentage of patients with a reduction in tumor burden (sum of partial responders + complete responders). Disease control rate was defined as a percentage of patients who achieved stable disease, partial response, and complete response to treatment.

### Blood collection and ctDNA analysis

Blood samples were collected using a EDTA vacutainer or cell-free DNA BCT tubes (Streck, La Vista, NE, USA). Plasma was separated from whole blood as previously described and stored at −80 °C. Cell-free DNA (cfDNA) was extracted from 3-5 mL of plasma using the QIAamp Circulating Nucleic Acid Kit (Qiagen, Hilden, Germany). The recovered cell free DNA samples were stored (−80 °C) until analysis. The ctDNA was quantified by droplet digital PCR (ddPCR). Trackable mutations for ctDNA were identified via standard pathology protocols from the molecular pathology report (BRAF mutant) or if BRAF wild type, using a customized melanoma NGS panel to sequence the patients tissue biopsy (Illumina, San Diego, CA, USA), as described by Calapre et al. (15). Commercially available and/or customized probes were used to analyze ctDNA by ddPCR as previously described (12, 15, 16). Mutational targets for ctDNA analysis are detailed in **Supplementary Table 1** (**Table S1** **).**

## Statistics

Descriptive statistics were used to analyze patient characteristics. Median OS and PFS were calculated using the Kaplan–Meier method and compared using the log-rank test. Association between ctDNA detectability at cessation and disease progression, with corresponding *p*-values, positive predictive values (PPV) and negative predictive values (NPV) were calculated using Fisher’s exact test. All statistical analyses were performed using GraphPad Prism version 9 (GraphPad Software Inc., San Diego, CA, USA) and SPSS version 25 (IBM, Armonk, NY, USA).

## Results

### Patient demographics

Overall, a total of 34 patients were identified who ceased ipilimumab and nivolumab for immune related toxicity and had ctDNA available for analysis (**Supplementary Figure 1**). Baseline demographics are represented in **Table 1**. Median age was 52 years (range: 20-86 yrs.) and 62% (n=21) were male. All patients had a good performance status (50% - ECOG 1). Over half of the cohort had a *BRAF* mutation, with 53% of the cohort having had at least one line of prior systemic therapy. Median number of cycles of ipilimumab and nivolumab were 3 (range: 1-4). Fourteen patients (41%) had two or greater high grade immune related adverse events, at least one of which led to discontinuation of treatment. There were 48 high grade irAE documented for 34 patients. The irAEs were predominantly colitis (n= 14) and hepatitis (n=21) but also included pneumonitis, nephritis, skin rash, hypophysitis, diabetes, pancreatitis and hematological toxicities. All patients received immunosuppression with steroids as treatment for immune related adverse events.

**Table 1:** Clinical characteristics at baseline (n=34).

Variable		Number (%)	
Age			
	Median	53	Range (20-86)
	<65	25	(74%)
	>65	9	(26%)
Sex			
	Female	13	(38%)
	Male	21	(62%)
AJCC stage			
	M1a	6	(18%)
	M1b	1	(3%)
	M1c	19	(56%)
	M1d	8	(23%)
ECOG			
	0	17	(50%)
	1	17	(50%)
Brain only mets - baseline			
	Yes	3	(9%)
	No	31	(91%)
Prior lines of treatment (ICI monotherapy, Targeted therapy, chemotherapy)			
	0 1	16 12	(47%) (35%)
	2	5	(15%)
	≥3	1	(3%)
Mutation status (tissue)			
	BRAF V600E/K	18	(53%)
	NRAS	4	(12%)
	Other	12	(35%)
High grade (III/IV) irAE			
	Single toxicity	20	(59%)
	≥2 toxicities	14	(41%)
Toxicity events (n=48)			
	Hepatitis	21	
	Colitis	14	
	Pneumonitis	1	
	Nephritis	1	
	Endocrine	2	
	Rash	4	
	Other	4	

### Response rates to treatment at the time of cessation for toxicity

The median time from last treatment to response assessment was 4.3 weeks (range: 0-16 weeks). The objective response rate (ORR) on first response assessment following discontinuation for the whole cohort was 76% (**Supplementary Table S2**). The disease control rate (DCR) was 85%. The ORR was non significantly higher in treatment naïve patients (n=16, 88%) compared to pre-treated patients (n=18, 67%).

**Figure 1:**
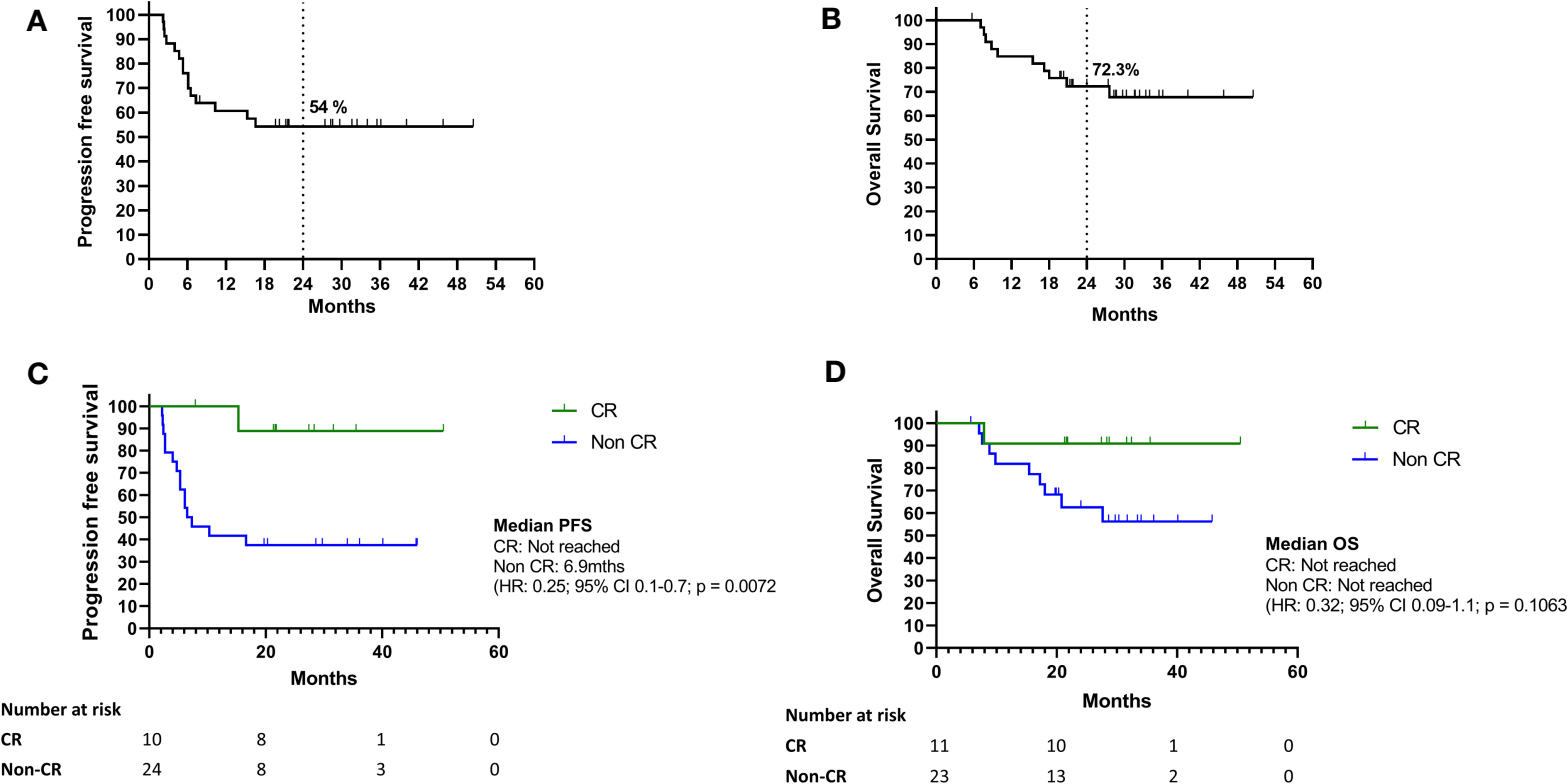
**(A–D)** Survival outcomes of study cohort. **(A–D)** Kaplan Meier curves for progression free survival and overall survival of the whole cohort **(A)** and according to response at first response assessment after treatment cessation for toxicity **(B)**. Kaplan Meier curves for progression free survival **(C)** and overall survival **(D)** for BOR between CR and non-CR (CR, complete response; non-CR, partial response; SD, stable disease; PD, Progressive disease).

Median PFS and OS have not been reached for the whole cohort (**Figures 1A, B**); at 2 years, the PFS rate was 54% and OS rate was 72.3%. The median PFS for patients with CR was not reached, whereas for patients with non-CR it was 6.9 months (PR: 26.7 months, SD: 16.6 months and PD: 2.4 months) (**Figure 1C**). Median OS was not reached in all patients who gained benefit from treatment (CR/PR/SD) and 18 months in patients who had disease progression on their first response assessment (**Figure 1D** **).**

Disease progression occurred in 16 of 34 patients, with disease progression at first response assessment in 5 patients and confirmed on repeat imaging after 4-12 weeks. No pseudoprogression was identified in this cohort. For patients that had non-CR clinical benefit (PR and SD) at first response assessments, 11 of 18 (61%) went on to develop disease progression during follow up. Only 1 of 11 (9%) patients in the CR group developed disease progression. A total of 10 deaths occurred, all related to disease progression. There were no deaths due to immune toxicity.

All the patients stopped the ICI combination regime and were not rechallenged with ipilimumab and nivolumab. However, 23 of 34 (68%) patients went on to be re-challenged with single agent nivolumab after recovering from toxicity. Four of these patients with partial response at time of treatment cessation due to toxicity achieved complete response after resumption of single-agent ICI. (**Supplementary table 3**). Of note, out of the 11/34 patients not rechallenged with single agent nivolumab following discontinuation, five of these patients did not resume treatment because of disease progression on their first response assessment.

Half of the patients received more than 2 cycles of combination ICI before stopping treatment due to toxicity. The number of cycles delivered prior to treatment cessation demonstrated a trend towards improved PFS, but this was not statistically significant (**Supplementary Figure 2**). The median PFS for the patients with less than or equal to two cycles was 10.3 months and not reached in the patients who received three or more cycles of treatment (HR: 3.22, 95% CI 1.2-8.9, p= 0.0633).

### Rechallenge vs no rechallenge

Excluding patients with initial disease progression, resumption rate of immunotherapy for patients with an initial complete response was 64% (n=7/11) versus 89% for those with non-CR (PR and SD, n=16/18). Despite this, the eventual disease progression rate was higher in the non-CR group (56%) compared to the CR group (9%). Of the 23 patients that were rechallenged with nivolumab, 11 (48%) had a flare of immune related toxicity following treatment. None of the patients with disease progression on first response assessment were rechallenged with nivolumab monotherapy.

In the group with initial CR, PR or SD who ultimately developed disease progression, the majority of patients (9/11) were rechallenged with single agent immunotherapy after recovering from the initial immune related toxicity. Three of these nine patients (33%) experienced a second flare in the immune related adverse event necessitating further immunosuppression and treatment delays. Of the patients with detectable ctDNA at cessation of combination immunotherapy (2=SD, 5=PD), only 1 patient was rechallenged. The patient’s ctDNA kinetics demonstrated a persistent rise during rechallenge with immunotherapy preceding radiological confirmation of progression by 5 months (**Supplementary Figure 3**).

### Detection of ctDNA at cessation of treatment due to toxicity predicts progression

**Figure 2:**
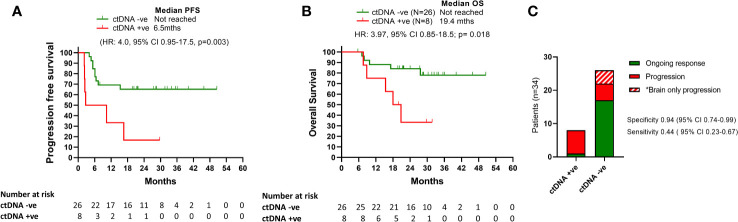
Survival outcomes relative to ctDNA detection. **(A)** and **(B)** Kaplan Meier curves for PFS and OS according to ctDNA being detectable or undetectable at time of treatment cessation due to toxicity. **(C)** Bar graph representation of patients ctDNA detection status at the time of treatment cessation relative to disease progression.

We measured ctDNA levels in plasma collected at the time or within 6 weeks of treatment cessation due to toxicity. The median time between last dose of the combination treatment and plasma collection was 2.1 weeks (range: -3 to 6 weeks). Detectable ctDNA was associated with shorter PFS and OS (**Figures 2A, B**). Median PFS for patients with detectable ctDNA from plasma collected at time of toxicity was 6.5 months while not reached for patients with undetectable levels (HR: 4.0, 95% CI 0.95-17.5, p=0.0023). Median OS for patients with detectable ctDNA at cessation for toxicity was 19.4 months and not reached for undetectable ctDNA (HR: 3.9, 95%CI 20.8-18.6, p=0.024).

**Figure 3:**
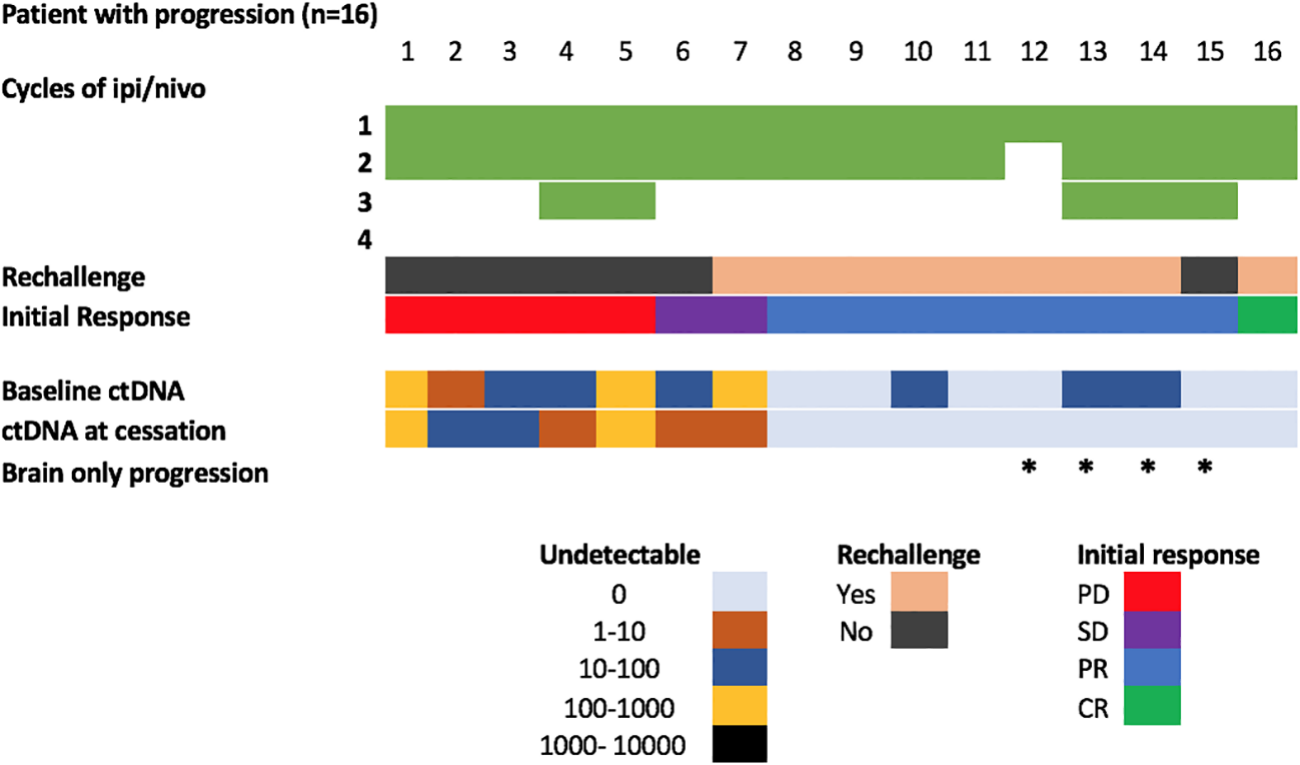
Overview of patients with disease progression during follow-up. * Represents patients with brain only progression.

Positive ctDNA at the time of cessation was highly specific for predicting progression (specificity 0.94, 95% CI 0.74-0.99, positive predictive value 0.88, 95% CI 0.53-0.99), with 7/8 patients with detectable ctDNA at the time of toxicity developed PD at follow up (median time to progression: 6.5 months; Range: 2.2-16 months) (**Figure 2C**). Of the 7 patients with detectable ctDNA at time of treatment cessation for immune related toxicity, 5 had concordant primary PD on the corresponding first response assessment (**Figure 3**). The other two patients had stable disease, with one of them resuming single agent nivolumab, but ultimately both progressed during follow up.

In contrast, 9/26 patients (34%) with undetectable ctDNA at the time of treatment limiting toxicity developed disease progression (median time to progression: 6 months, range: 4.0-15.3 months). Thus, despite its association with improved survival, ctDNA negativity was not a strong predictor of ongoing disease control (sensitivity 0.437, 95% CI 0.23-0.67). Notably, 4/9 (44%) ctDNA negative patients who had disease progression had brain only disease progression.

## Discussion

This study confirms that ipilimumab and nivolumab for advanced melanoma is an effective treatment in the real-world population. In this cohort of patients who stopped treatment for toxicity, the survival outcomes do not seem to be compromised. Our results underscore that detectable ctDNA around the time of treatment cessation due to toxicity is highly predictive of resistance to treatment and disease progression regardless of the initial imaging response (**Figures 2A, B**).

Plasma ctDNA concordance with both imaging and tumor burden has been established ( (11). However, its application into clinical practice has been limited by its reduced sensitivity. Our results mirror other ctDNA studies in melanoma which report a high specificity but low sensitivity (9, 17, 18). In our cohort, 9/16 (56%) of patients who progressed had undetectable ctDNA at the time of treatment cessation for toxicity. Notably, 4 of the 9 false-negative cases had intracranial disease only, which is known to not be detectable by ctDNA (19). Moreover, false negatives may be also related to low disease burden, prior BRAF inhibitor treatment (33%), or sampling error (10, 20).

The results of this retrospective cohort analysis of real-world patients treated with combination immunotherapy for metastatic melanoma demonstrate outcomes comparable to those seen in the pivotal CheckMate 067 trial (21). Despite having a significant proportion of patients treated with prior therapy and experiencing significant immune related toxicity, this group had excellent response rates, prolonged PFS and OS with flattening of the survival curves at the two-to-three-year time point, with median OS not yet reached. The first response assessment demonstrating complete response (CR) predicted for improved PFS and OS compared to non-CR (SD, PR). The objective response rates seen in our cohort were high (76%) compared to CheckMate 067 and other real world reports (30-58%). This is likely due to the small numbers, selection bias and the high use of PET imaging in our cohort.

Discontinuation or treatment with immunosuppressants due to toxicity was seen in over one third of patients in CheckMate 067 (1) and even higher outside of clinical trials (40-60%) (22, 23). Toxicity and subsequent discontinuation or delays in the reintroduction of immunotherapy is common (1, 23). Retrospective analysis has shown that neither permanent discontinuation of immunotherapy nor exposure to steroids leads to compromised outcomes (24–26). In our cohort there was a trend towards shortened PFS and OS in those that received less than two cycles. This has also been reported by Asher et al. who found that patients who had > 2 cycles had a statistically significant improvement in overall survival (HR: 0.35, 95% CI 0.18-0.68, p=0.002) (22). It has recently been reported that early steroid use is associated with reduced efficacy and poorer long-term outcomes (27). In the current treatment landscape, steroid use is not optional in the context of high grade immune related toxicity and is therefore not modifiable in this cohort, however work is being done to mitigate immune toxicity and minimize steroid exposure (28).

The optimal duration of immunotherapy remains unknown. In the neo-adjuvant melanoma space, it is becoming apparent that a short exposure of immunotherapy can lead to deep and durable responses (29). This may also be relevant to patients in the advanced setting who stop treatment for toxicity and attain early CR. In our cohort, for selected patients with a complete metabolic response on PET imaging at first response assessment after toxicity and undetectable ctDNA, the risk of recurrence was less than 10%. Although the rate of resumption of immunotherapy after toxicity was lower in those patients with CR on imaging, it still occurred in >60% of this cohort. With a risk of flaring immune toxicity of up to 50% in our cohort, it is doubtful that rechallenge is beneficial in this group.

The risk of flare of toxicity with rechallenge of single agent CPI in our cohort was 50% and this is consistent with the literature (20-50%) (30, 31). Currently, there is no clear biomarker to determine whether those with SD, PR, or CR benefit from re-challenge with nivolumab monotherapy following discontinuation of the combination for significant toxicity. No clear risk factor to identify those at risk of flare of irAE following re-challenge have been identified. In ASCO and ESMO immunotherapy guidelines the recommendation is that grade IV irAEs warrant permanent discontinuation. ASCO guidelines go further to recommend permanently stopping immunotherapy for certain grade III toxicities such as myocarditis, nephritis, hepatitis, neurological toxicities and pneumonitis (32, 33). These guidelines recommend if there is evidence of response, that resumption of therapy may not be advisable but if there is inadequate radiological response, consideration should be given to resuming immunotherapy. In our cohort, patients who had CR on initial response imaging after stopping for toxicity had a very low rate of disease progression regardless of whether they received further immunotherapy or not.

We propose that ctDNA could be a useful biomarker in this situation to assist in determining the value of restarting treatment and have devised a decision tree that may assist in determining who would benefit from rechallenge with the risk of recurrence of toxicity (**Figure S4**). This cohort has demonstrated that undetectable ctDNA and CR on imaging after stopping immunotherapy for toxicity results in high rates of long-term durable control. It appears in this small group that rechallenge does not impact outcome. For patients with immunotherapy related toxicity, who have persistent ctDNA at 8 – 12 weeks, rechallenge with immunotherapy needs to be monitored very closely as the risk of disease progression is significant (10, 15). Such a decision tree would need to be validated in larger prospective trials, but our retrospective study gives credence to the fact that this, once validated, may be a feasible approach to assist clinicians in deciding whom to rechallenge.

Overall, this study adds to our previous reports of ctDNA to serve as highly specific marker of progression in patients undergoing treatment cessation (34). Moreover, it corroborates the PFS and OS benefit associated with undetectable ctDNA in melanoma patients treated with immunotherapy, as previously reported (12, 14, 35–39). Due to the small numbers of cases, we cannot draw robust conclusions about the value of plasma ctDNA at the time of treatment cessation due to irAEs above the current multifactorial decision making utilized by clinicians to assess the risk-benefit ratio of restarting immunotherapy. Furthermore, we could hypothesize that patients with complete response and negative ctDNA at 12 weeks could stop treatment, regardless of toxicity. At the current time, the low sensitivity of ctDNA prevents its use in monitoring minimal residual disease. However, our results support the need for larger studies exploring this provocative hypothesis.

## Data availability statement

The raw data supporting the conclusions of this article will be made available by the authors, without undue reservation.

## Ethics statement

The studies involving humans were approved by Human Research Ethics Committee of Edith Cowan University (Nos. 11543 and 18957) and Sir Charles Gairdner Hospital (No. 2013–246 and RGS0000003289). Written consent was obtained from all patients under approved human research ethics committee protocols that complied with the Declaration of Helsinki. The studies were conducted in accordance with the local legislation and institutional requirements. The participants provided their written informed consent to participate in this study.

## Author contributions

LW: Conceptualization, Data curation, Formal analysis, Funding acquisition, Investigation, Methodology, Validation, Writing – original draft, Writing – review & editing. AR: Data curation, Investigation, Methodology, Project administration, Writing – review & editing. BA: Formal analysis, Funding acquisition, Validation, Writing – review & editing. LC: Conceptualization, Data curation, Formal analysis, Investigation, Supervision, Validation, Visualization, Writing – review & editing. MM: Conceptualization, Funding acquisition, Investigation, Supervision, Visualization, Writing – review & editing. EG: Conceptualization, Data curation, Formal analysis, Funding acquisition, Methodology, Project administration, Resources, Software, Supervision, Validation, Visualization, Writing – review & editing.
