# Supplementary material for: Detectable ctDNA at the time of treatment cessation of ipilimumab and nivolumab for toxicity predicts disease progression in advanced melanoma patients
Source: Front Oncol. 2023 Dec 19;13:1280730. doi: 10.3389/fonc.2023.1280730 (PMC10766351; doi:10.3389/fonc.2023.1280730)
Supplement: Supplementary file 1 [file DataSheet_1.pdf]

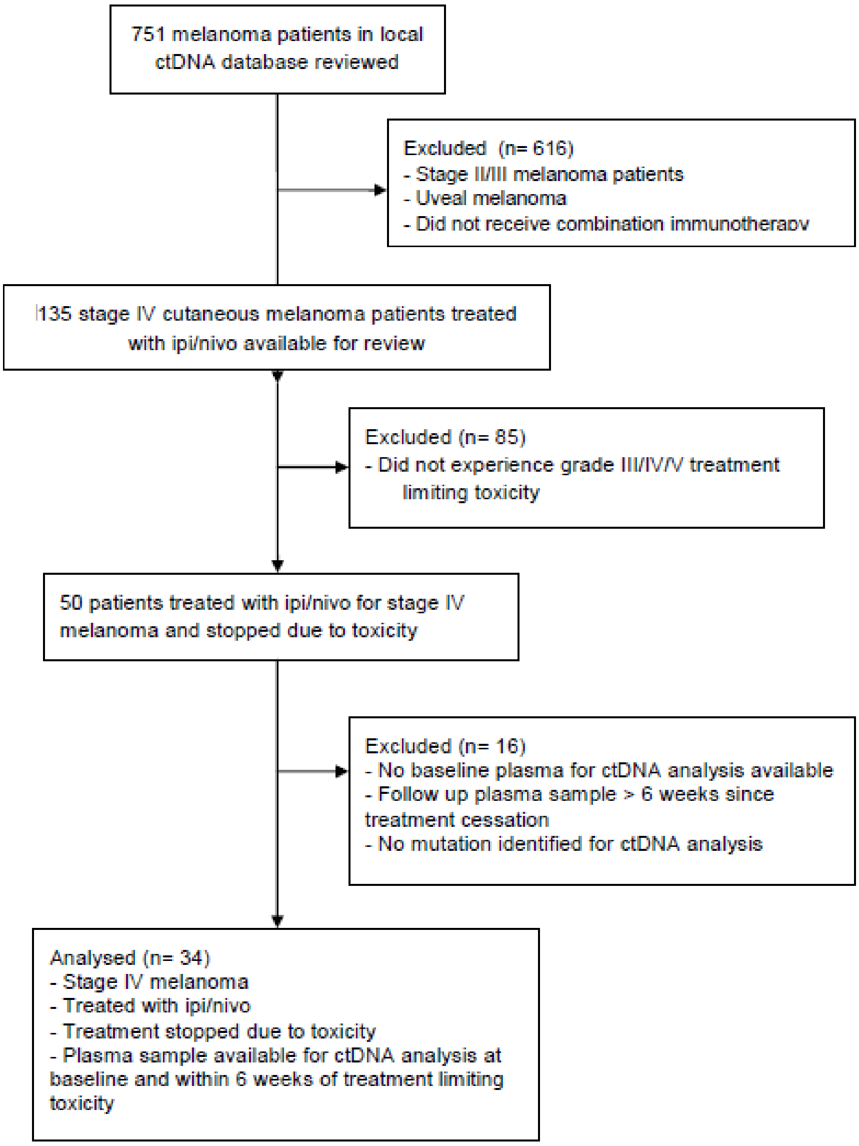

Figure S1: Consolidated Standards of Reporting Trials (CONSORT) flow diagram. Flow diagram depicts the disposition of patients for the study. Reasons for exclusion were no grade III or more toxicities, no plasma collected prior commencing treatment (baseline) or within 6 week of treatment cessation, or no mutation identified for ctDNA monitoring.

Table S1 – Detailed list of mutation types identified and analysed by ddPCR and ctDNA status at cessation of therapy for toxicity

**Mutations detected**	**Number of Patients**	**ctDNA status of patients at treament cessation**	
		ctDNA +ve	*ctDNA -ve*
BRAF			
BRAF p.V600E	17	4	13
BRAF p.V600K	1	1	
BRAF p.G615V	1		1
BRAF p.K601E	1		1
NRAS			
NRAS p.Q61K	2		2
NRAS p.Q61R	1		1
NRAS p.Q61H	1		1
**Other**			
TERT C250T	5	2	3
GNAQ p.Q209L	2		2
PIK3CA p.(Glu545Lys)	1		1
KRAS p.Q61K	1	1	
KITp.A502Y	1		1

Table S2. Efficacy assessment at time of treatment cessation

**Response**	**Whole Cohort (n=34)**	**Treatment naïve (n=16)**	**Pre-treated (n=18)**
CR	11 (32%)	4 (25%)	7 (39%)
Non-CR			
PR	15 (44%)	10 (63%)	5 (28%)
SD	3 (9%)	1 (6%)	2 (11%)
PD	5 (15%)	1 (6%)	4 (22%)

Best Initial Response (n = 34)	Rechallenge (n= 23)	No rechallenge (n=11)	Disease progression - after initial response (n= 11)
CR (n=11)	7 (64%)	4 (36%)	1 (9%)
Non-CR (n= 23)			
PR (n= 15)	14 (93%)	1 (7%)	8 (53%)
SD (n=3)	2 (67%)	1 (33%)	2 (66%)
PD (n= 5)	0	5 (100%)	N/A

Table S3. Initial response on imaging following cessation of therapy due to toxicity, rates of rechallenge with nivolumab and disease progression across the response categories

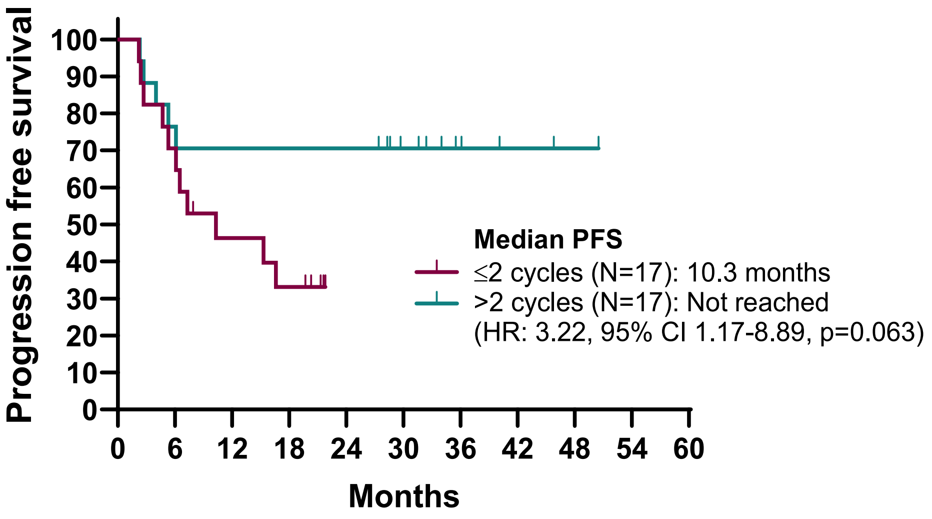

Figure S2. Kaplan-Meier curves depicting PFS according to number of cycles of combination immunotherapy received prior to cessation due to toxicity.

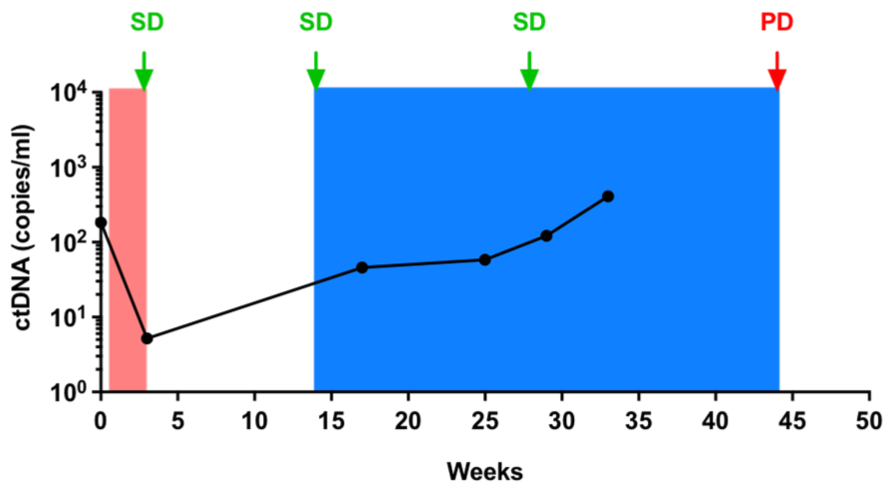

Figure S3- (PID 1126) Longitudinal tracking of ctDNA during combination immunotherapy (red) and on rechallenge with nivolumab (blue) following recovery from irAE. Disease assessments by radiological imaging are indicated by arrows and labelled as *SD* stable disease, and *PD* progressive disease.

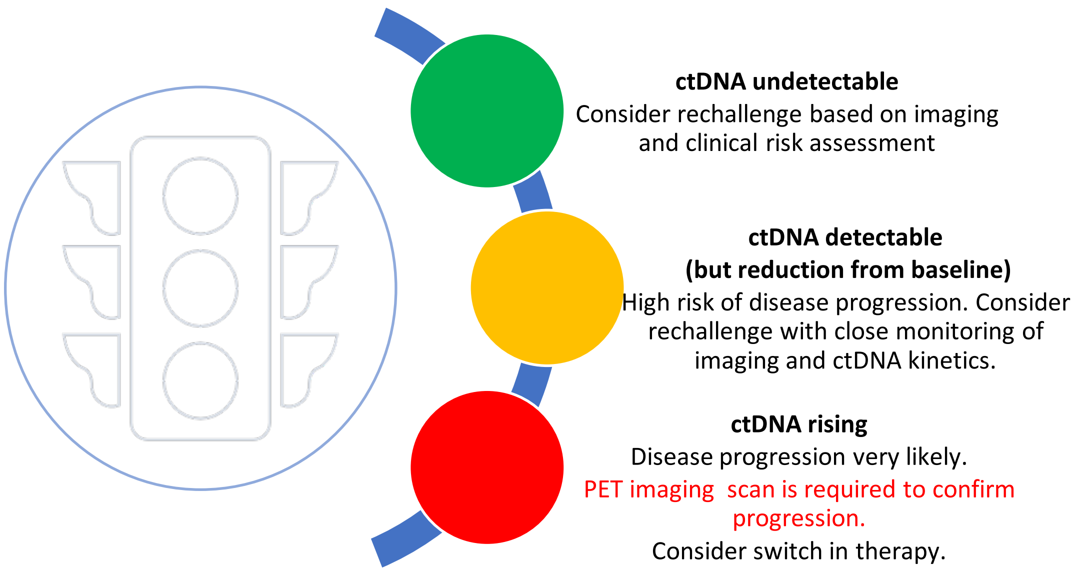

Figure S4. Proposed incorporation of ctDNA into decisions about rechallenge with immunotherapy following cessation of ipilimumab/nivolumab for treatment limiting immune related adverse events.
